# Case Report: Two cases of recurrent syncope caused by *KCNH2* gene mutation in congenital long QT syndrome

**DOI:** 10.3389/fcvm.2026.1778685

**Published:** 2026-04-29

**Authors:** Aihua Xing, Lifang Liu, Wenjuan Pei, Xiaoyan Zhang

**Affiliations:** 1 Fenyang Hospital, Department of Laboratory Medicine, Shanxi University of Medicine, Shanxi, China; 2 Graduate School, Shanxi Medical University, Shanxi, China

**Keywords:** *KCNH2*, long QT syndrome, LQTS, QT prolongation, syncope

## Abstract

This study presents two cases of congenital long QT syndrome caused by *KCNH2* gene mutations. It highlights the critical role of genetic testing in its diagnosis and underscores the importance of early detection and personalized treatment strategies to enhance patient outcomes. Comprehensive clinical data were collected from the patients, including measurements of the QT interval and calculations of the corrected QT interval (QTc) using the Bazett correction formula. Genetic testing identified heterozygous pathogenic variants in the *KCNH2* gene in both patients. The variant in Case 1 was a frameshift mutation (c.234_241dup, p.Gln81Leufs*38), located in exon 2 (NM_000238.4). Case 2 carried a missense mutation (c.1841C > T, p.Ala614Val) in exon 7 (NM_000238.4). Predictions from multiple bioinformatics tools were consistent with the pathogenicity of both variants. The findings underscore that early diagnosis and tailored therapeutic approaches are essential for improving the prognosis of individuals with long QT syndrome. It is imperative for clinicians to increase awareness of long QT syndrome, particularly in patients presenting with syncope as an initial symptom, to inform clinical diagnosis, treatment, and prognostic assessment.

## 1 Introduction

Long QT syndrome (LQTS), also referred to as delayed repolarization syndrome, can be categorized into acquired LQTS and congenital LQTS. Congenital LQTS is linked to gene mutations, specifically those affecting genes that encode ion channel proteins in cardiac muscle cell membranes. These mutations can result in ion channel dysfunction within the heart muscle, thereby causing abnormal repolarization (1). LQTS currently comprises 17 subtypes. Among these, mutations in *KCNQ1*, *KCNH2*, and *SCN5A*—causing LQT1, LQT2, and LQT3, respectively—are well-established as pathogenic subtypes (2, 3). In contrast, the pathogenic evidence for genes such as *AKAP9, ANK2, CAV3, KCNE1, KCNE2, KCNJ2, KCNJ5, SCN4B*, and *SNTA1* remains limited or controversial. *CALM1-3* and *TRDN* show strong associations with LQTS, particularly when accompanied by atypical manifestations such as neonatal atrioventricular block (4), while *CACNA1C* is supported by moderate pathogenic evidence (5). Genetic testing serves as a crucial tool for the diagnosis and classification of LQTS. Congenital LQTS is predominantly inherited in an autosomal dominant fashion without congenital deafness, known as Romano-Ward syndrome, whereas autosomal recessive inheritance is frequently associated with congenital deafness, termed Jervell and Lange-Nielsen syndrome (2, 3). Acquired LQTS is associated with electrolyte imbalances, such as hypokalemia, hypocalcemia, and hypomagnesemia (6); certain medications, including antiarrhythmic drugs like amiodarone (7) and opioid analgesics (8); as well as cardiac conduction block and heart disease (9). LQTS often presents without overt clinical symptoms, yet it poses a significant risk for fatal arrhythmias (10), which can result in syncope or sudden cardiac death, particularly in adolescents, where it is the leading cause of sudden death. In individuals with LQTS, syncope frequently represents the initial clinical manifestation (2). The arrhythmias associated with congenital LQTS predominantly involve ventricular tachyarrhythmias, such as polymorphic ventricular tachycardia. A minority of patients may display atypical characteristics, including atrioventricular block and atrial arrhythmias (3). Electrocardiographic findings typically reveal a prolonged QT interval and abnormal T or U waves, predisposing patients to life-threatening Torsade de Pointes (TdP) and sudden cardiac death (10). Notably, over one-third of mutation carriers exhibit normal QT intervals on resting ECGs (11), complicating the diagnosis of LQTS. The advent and widespread use of genetic testing have enhanced the detection rate of LQTS and have shifted the focus towards understanding its pathogenic mechanisms. In this study, we report the clinical manifestations, auxiliary examinations, and genetic test results of two diagnosed with LQTS, caused by pathogenic variants in the *KCNH2* gene. Our findings aim to serve as a reference for the clinical diagnosis, treatment, and prognostic evaluation of LQTS patients.

## 2 Case presentation

### 2.1 Case 1

**Figure 1:**
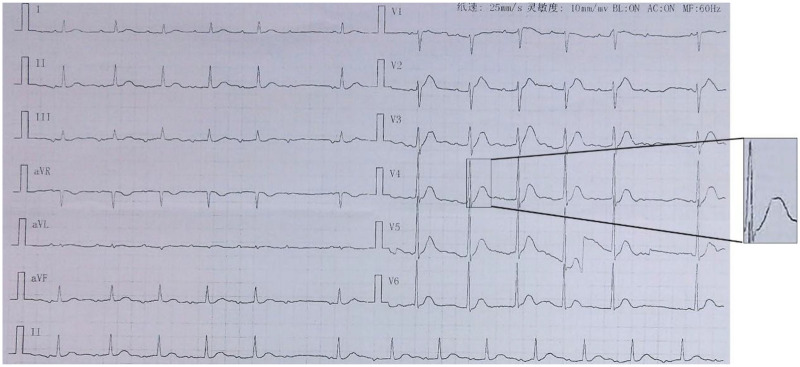
Case1 ECG on the day of admission to the local hospital shows sinus rhythm 83 bpm, QT/QTc 338 ms/397 ms, second-degree AV block, type I.

**Figure 2:**
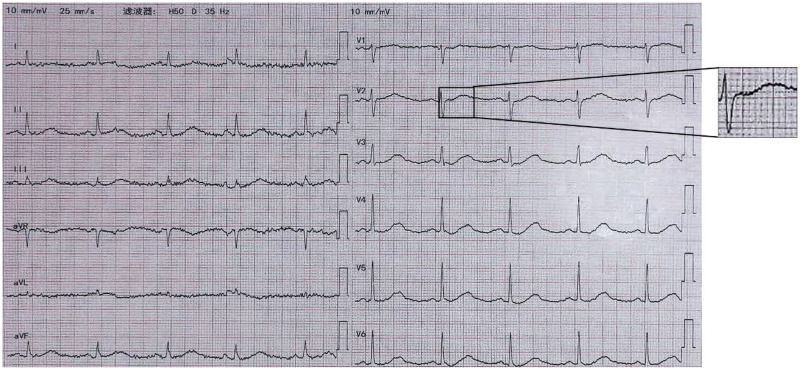
Case1 emergency electrocardiogram shows sinus rhythm 59 bpm, QT/QTc 518 ms/518 ms.

**Figure 3:**
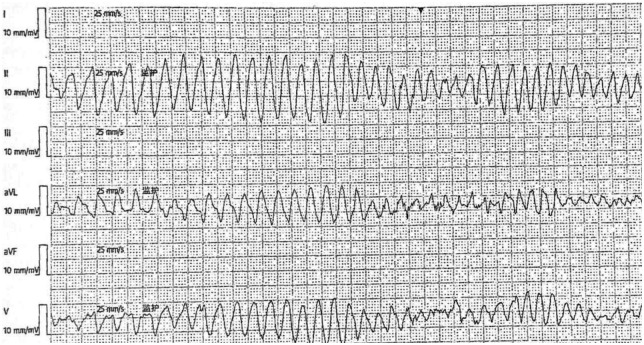
Case 1 dynamic ECG during inpatient syncopal episode (Torsade de Pointes).

A 64-year-old female patient was admitted to the hospital presenting with a history of “intermittent syncopal episodes over the past two years and recurrent episodes over the preceding two days”. Two years prior, the patient began experiencing transient episodes of loss of consciousness, accompanied by urinary incontinence and twitching of the hands and feet. Each episode lasted about one minute, after which she regained consciousness spontaneously and experienced no discomfort. At that time, she presented it to a local hospital, where an electrocardiogram (ECG) revealed sinus bradycardia; however, this finding was not subsequently addressed. Two days prior to the current admission, in the afternoon, the patient experienced a sudden onset of syncope while seated, without any apparent precipitating factors. This episode was characterized by a brief loss of consciousness lasting approximately one minute, after which the patient regained full consciousness spontaneously and reported no residual discomfort. On the morning of the day of admission, around 8 a.m., the patient experienced another episode, this time accompanied by urinary incontinence and limb convulsions. Notably, there were no signs of upward eye rolling, oral foaming, or tongue biting. The patient was promptly admitted to a local hospital. (Figure 1) Emergency ECG showed sinus rhythm (83 bpm) and second-degree AV block, type I. Following approximately 10 min of intravenous fluid resuscitation, the patient regained consciousness without any accompanying symptoms such as chest tightness, shortness of breath, chest pain, palpitations, or limb numbness/weakness. Upon transfer to our hospital's emergency department, laboratory tests (including electrolytes, complete blood count, and renal function) and head CT demonstrated no significant abnormalities except for mild hypokalemia (3.43 mmol/L). (Figure 2) The ECG showed sinus bradycardia at a rate of 59 bpm with a prolonged QTc interval (518 ms) and second-degree type I AV block. After consultation with our department, the patient was admitted to the hospital for further investigation of the cause of syncope. Since the onset of symptoms, the patient has been in poor spirits, with a poor appetite but normal sleep and bowel movements. The electrocardiogram recorded during the patient's syncopal episode in the hospital, as captured by ambulatory electrocardiography, is shown in (Figure 3).

**Figure 4:**
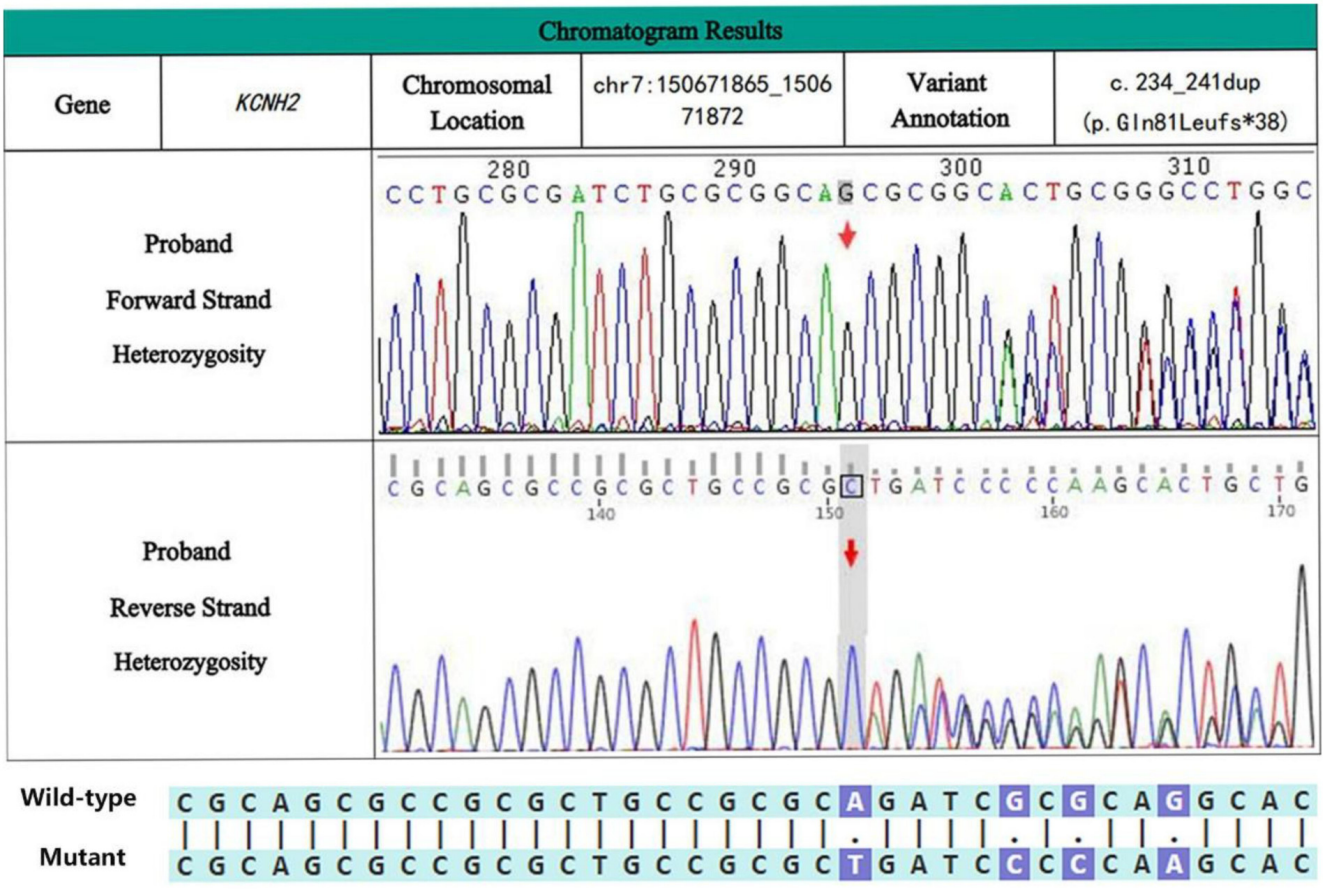
Sanger sequencing map of case 1 patients.

Upon admission, the patient underwent a comprehensive series of pertinent laboratory tests and examinations. Subsequent genetic testing identified a heterozygous variant in the *KCNH2* (NM_000238.4) gene, specifically a mutation located at exon 2, c.234_241dup, resulting in the amino acid alteration p.Gln81Leufs*38 (Figure 4). The patient received treatment that included cardiac monitoring, rhythm regulation, lipid-lowering therapy, and additional symptomatic supportive interventions. The discharge diagnoses included LQTS, cardiac arrhythmia, second-degree type I atrioventricular block, sinus bradycardia, atrial premature contractions (atrial extrasystoles), paroxysmal atrial tachycardia, ventricular premature contractions, electrolyte disturbance, and hypokalemia. Upon discharge, the patient was instructed to continue taking Shensong Yangxin Capsules as prescribed. The option of elective dual-chamber implantable cardioverter-defibrillator (ICD) implantation was also discussed.

One month later, the patient successfully underwent the implantation of a biventricular ICD. The operation went smoothly, and the incision healed well after the operation. After discharge from the hospital, he was advised to take medication regularly, and the remaining sutures were removed after 3–5 days. She was rechecked in the outpatient clinic after 1 month and followed up for any discomfort.

### 2.2 Case 2

**Figure 5:**
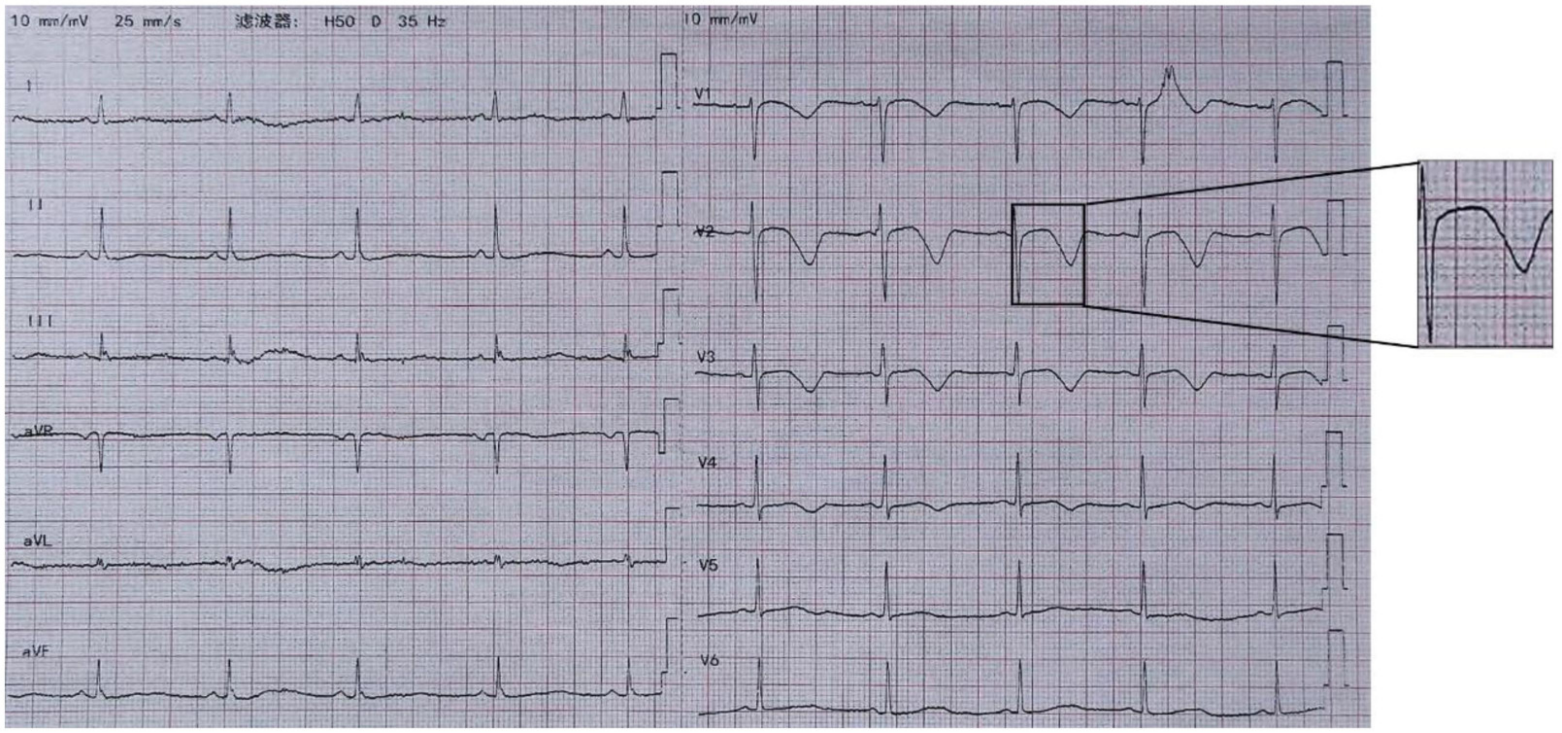
Case 2 ECG of the patient on the day of admission shows sinus rhythm 61 bpm, QT/QTc 528 ms/531 ms.

A 14-year-old female patient was admitted to the hospital presenting with a history of “intermittent syncopal episodes over the past 8 years, with exacerbation over the preceding 2 days”. Eight years prior to this admission, while sitting quietly, the patient developed dizziness and palpitations followed by syncope, characterized by loss of consciousness, unresponsiveness, clenched fists, and limp limbs, without associated dyspnea, convulsions, frothing, or incontinence. She reportedly regained consciousness spontaneously after several minutes of stimulation to her philtrum and Hoku point by family members, with no residual symptoms. Four years earlier, she had been diagnosed with LQTS, Patent Ductus Arteriosus (PDA), and ventricular arrhythmia at an outside hospital. At that time, surgical intervention was deemed unnecessary, and she was managed with regular outpatient medication. Two days prior to admission, the patient's dizziness worsened and was accompanied by chest tightness. At 10 PM, she suddenly lost consciousness and became unresponsive. Notably, there were no associated convulsions, frothing at the mouth, or urinary incontinence. Family members stimulated her philtrum, and she regained consciousness after several minutes, remaining alert and oriented thereafter. She was subsequently brought to our hospital for evaluation. During the emergency department evaluation, she suffered a recurrent syncopal episode accompanied by ventricular fibrillation (VF), as evidenced by ECG. After several minutes of external chest compression, sinus rhythm was restored, and the patient regained consciousness. She was then admitted to our department for further management. Emergency ancillary testing included an ECG, which revealed sinus rhythm at 61 bpm, a normal cardiac axis, and T-wave inversions in leads V1-V4 on the 18-lead ECG (Figure 5), with a prolonged QTc interval of 531 ms (reference range: <450 ms). Laboratory analyses indicated approximately normal blood counts, renal function, and coagulation profiles, with a noted decrease in potassium ion concentration (3.59 mmol/L) and normal levels of N-terminal B-type natriuretic peptide precursor. Upon reviewing her family history, it was discovered that her mother had experienced recurrent fainting spells and was diagnosed with “epilepsy” at another hospital before suffering sudden death at age 26.

**Figure 6:**
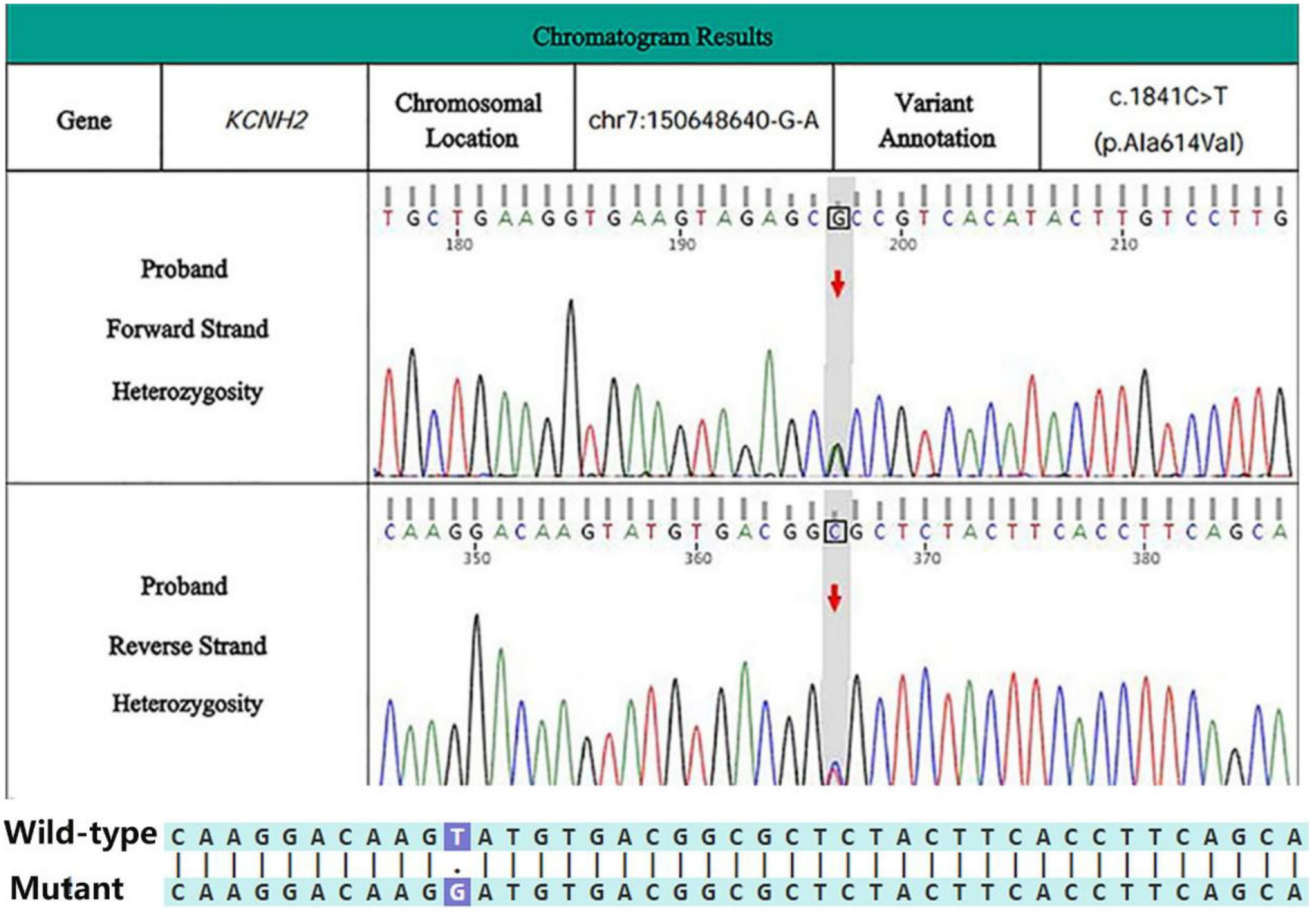
Sanger sequencing map of case 2 patients.

The initial clinical diagnosis included LQTS, VF, frequent ventricular premature contractions, cardiogenic syncope, PDA, electrolyte disturbance, and hypokalemia. Upon admission, the patient received treatment comprising antiarrhythmic therapy, potassium and magnesium supplementation, fluid replacement, nasal hemostasis, and anti-infective measures. Additionally, an ICD was implanted. Whole-exome sequencing identified a heterozygous variant in the KCNH2 (NM_000238.4) gene. The mutation site is located in the seventh exon at c.1841C > T, resulting in an amino acid change of (p.Ala614Val) (Figure 6).

**Figure 7:**
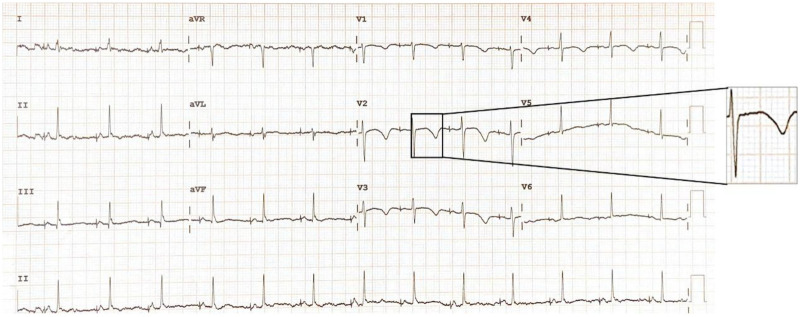
Case 2: recent follow-up ECG shows sinus rhythm 80 bpm, QT/QTc 452 ms/522 ms.

Following discharge, the patient was advised to change the medication at the incision site for three days, adhere to a regular medication regimen (Shensong Yangxin Capsule, Coenzyme Q10, Potassium Aspartate and Magnesium Aspartate Tablets, Propranolol Hydrochloride Tablets), and undergo regular electrocardiogram assessments at the cardiology clinic. The results of the recent follow-up electrocardiogram are shown in (Figure 7).

## 3 Discussion

We present two cases of LQTS caused by mutations in the *KCNH2* gene. The QT interval represents the duration from the onset of the QRS complex (Q wave) to the termination of the T wave, indicating the time required for ventricular depolarization and repolarization, which corresponds to the duration of the ventricular action potential (3). To mitigate the influence of heart rate on the QT interval, the corrected QT interval (QTc) is utilized, calculated using the Bazett formula.

The diagnosis of LQTS is based on ECG findings (QTc prolongation), family history, unexplained syncope, and genetic testing. A definitive diagnosis is made if any of the following criteria are met: (1) QTc ≥480 ms on repeated 12-lead ECG; (2) Schwartz score ≥3.5; or (3) identification of a pathogenic mutation, even with a normal QTc. Additionally, LQTS should be considered in patients with unexplained arrhythmic syncope and a QTc between 460 and 480 ms, after excluding other causes (12). According to ACMG standards, the variants carried by both patients were classified as “pathogenic,” further supporting the diagnosis.

In case 1, the QTc interval was within the normal range at the initial emergency presentation. The QT interval is inherently dynamic, varying considerably with genotype, heart rate, autonomic tone, and environmental triggers, including drugs and electrolyte disturbances (13). Approximately 20% of long QT syndrome patients demonstrate normal QTc on resting ECG—a phenomenon termed “electrocardiographically concealed LQTS” (14). *KCNH2* mutation carriers (LQT2) particularly show variable QTc expression; prior normal measurements do not exclude future risk. Notably, tetrahydrocannabinol (THC) exposure has precipitated Torsade de Pointes in *KCNH2* carriers, illustrating how genetic vulnerability may remain silent until specific environmental triggers intervene (15). Similarly, fever, electrolyte disturbances, and QT-prolonging medications can unmask concealed LQTS and trigger malignant arrhythmias (6, 16). Consequently, isolated resting QTc assessment cannot reliably identify all at-risk individuals; comprehensive evaluation incorporating dynamic monitoring, environmental factors, and genetic background is essential.

Current therapeutic approaches for LQTS primarily involve pharmacological interventions. Beta-blockers are the preferred first-line treatment and are effective in reducing the risk of arrhythmias (17). Notably, nadolol is the only beta-blocker that significantly diminishes the incidence of cardiovascular events in LQTS type 2 (18, 19). However, beta-blockers should be avoided in patients with specific contraindications, such as severe asthma, bradycardia, and atrioventricular nodal block (18). The protective mechanism of beta-blockers against LQTS is to slow down the heart's response to adrenaline induced by exercise or stress, slowing down the heart rate and thus reducing the risk of tip-twist ventricular tachycardia. Second, lifestyle modification. Patients should try to avoid factors that may induce arrhythmias due to strenuous exercise, emotional excitement, and sudden noise stimulation (3), and avoid drugs that prolong the QT interval (5). Third, ICDs. ICDs are effective in preventing sudden death in patients at high risk of severe arrhythmias and poor drug therapy (2). Fourth, left cardiac sympathetic denervation (LCSD). By cutting or removing specific sympathetic ganglia, it increases the threshold of VF (17) and reduces the risk of arrhythmic episodes.

In this study, we conducted a retrospective analysis of two patients diagnosed with LQTS, both of whom shared a history of recurrent syncope episodes over several years, spontaneous recovery without sequelae, QT interval prolongation, electrolyte imbalances, and hypokalemia, and genetic testing confirmed that all cases were caused by mutations in the *KCNH2* gene. Both patients met the diagnostic criteria with Schwartz scores ≥3.5 (Case 1: 5; Case 2: 4.5), indicating a high likelihood of LQTS. It is crucial to accurately identify the etiology of syncope, as symptoms such as syncope and palpitations in LQTS can mimic epileptic seizures, leading to potential misdiagnosis as epilepsy. Previous literature has documented instances of LQTS patients being misdiagnosed with epilepsy (20).

Whole-exome sequencing of peripheral blood samples revealed that the first case involved an LQT2 patient carrying a c.234_241dup (p.Gln81Leufs*38) frameshift variant in the *KCNH2* gene. This variant, resulting from a non-triplet base repeat, may theoretically lead to loss of normal protein function through mechanisms such as nonsense-mediated mRNA decay (NMD) or premature termination of the amino acid sequence, thereby impairing normal protein function. This variant is not currently reported in the large-scale population frequency database gnomAD. Sanger sequencing validation suggests that case 1 patients are carriers of this heterozygous variant. The patient in Case 1 was subjected to genetic testing and diagnosed with type 2 LQTS, attributed to a mutation in the *KCNH2* gene. This diagnosis was corroborated by the additional information that the patient had a nephew who experienced sudden cardiac death. Mutation Taster software predicts this mutation to be pathogenic with a probability of 1 (https://www.genecascade.org/MutationTaster2021/).

Case 1 was diagnosed with late-onset LQT2 at age 64, resulting from an N-terminal frameshift mutation causing haploinsufficiency. She exhibited a concealed phenotype with normal or borderline QTc intervals but demonstrated heightened sensitivity to drugs (21). At an advanced age, a convergence of acquired factors—QT-prolonging medications, electrolyte imbalances, bradycardia, comorbidities, and postmenopausal loss of estrogen's cardioprotection (22)—triggered the expression of her latent genetic susceptibility. This case highlights the incomplete penetrance and variable expressivity of LQT2 (23), emphasizing that genetic causes should not be dismissed based on late presentation. Medication review and family screening are warranted.

Case 2 involves a patient carrying the A614V mutation in the *KCNH2* gene, a classic pathogenic variant located in the pore region of the hERG potassium channel. This mutation occurs in the connecting region between the second transmembrane domain (S5) and the pore domain (P-loop) (24) and exhibits a clear dominant-negative effect, leading to a significant reduction in the rapid delayed rectifier potassium current (IKr), thereby resulting in a severe arrhythmogenic phenotype (25, 26). Multiple bioinformatics tools indicate its deleterious nature: PolyPhen2 (http://genetics.bwh.harvard.edu/pph2/): This mutation is predicted to be probably damaging with a score of 1.000. Mutation Taster: Predicted as Deleterious. The CADD score for your variant is 26.2, indicating the mutation is “deleterious.” (https://cadd.gs.washington.edu/). Sanger sequencing validation confirms that case 2 is a heterozygous carrier of this variant. In clinical practice, to improve the diagnostic reliability of LQTS, patients with suspected LQTS should be screened for family members along with relevant genetic testing. Both patients underwent successful treatment with ICD implantation and were subsequently discharged.

## 4 Conclusions

In this study, we present the clinical manifestations, auxiliary examinations, and genetic test results of two patients diagnosed with LQTS. Our findings aim to serve as a reference for the clinical diagnosis, treatment, and prognostic evaluation of LQTS patients. LQTS is a complex and potentially fatal arrhythmic disorder that can manifest at any stage of life, thereby underscoring the importance of early diagnosis and intervention. The implantation of an ICD has proven effective in preventing malignant arrhythmias and sudden cardiac death. Following ICD implantation and pharmacological treatment, the patient achieved stabilization. It is imperative for healthcare professionals to heighten their awareness of LQTS and promptly identify patients exhibiting atypical or asymptomatic presentations to facilitate early intervention. Therapeutic strategies should be tailored according to the genetic subtype and clinical characteristics of the patients, with careful avoidance of medications that may prolong the QT interval, thereby averting severe outcomes. Furthermore, it is crucial to provide precise genetic counseling to patients and their families by elucidating the clinical features and genetic inheritance patterns of LQTS. This includes informing family members about the associated risks and advising them to undergo genetic screening and preventive measures. This variant is not currently documented in the large-scale population frequency database, gnomAD. Validation through Sanger sequencing indicates that the patient in Case 1 is a carrier of this heterozygous variant. In clinical practice, enhancing the diagnostic accuracy of LQTS necessitates screening suspected LQTS patients' family members in conjunction with appropriate genetic testing.

## Data Availability

The datasets for this article are not publicly available due to concerns regarding participant/patient anonymity. Requests to access the datasets should be directed to the corresponding author.
