# Supplementary figures and images for: Case Report: Two cases of recurrent syncope caused by KCNH2 gene mutation in congenital long QT syndrome
Source: Front Cardiovasc Med. 2026 Apr 29;13:1778685. doi: 10.3389/fcvm.2026.1778685 (PMC13170101; doi:10.3389/fcvm.2026.1778685)

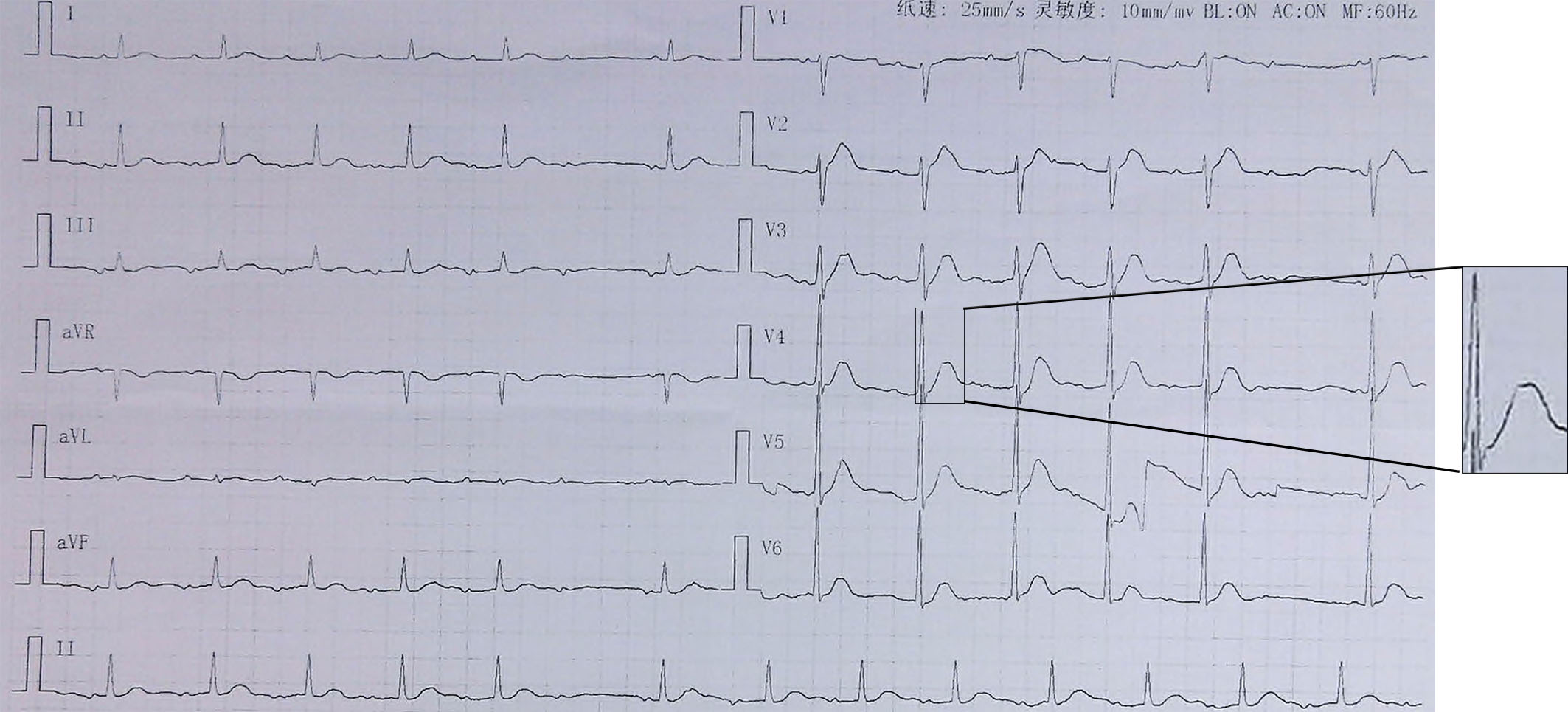

Supplement: Supplementary file 1 [file Datasheet1.zip › 图片/Figure 1.jpg]

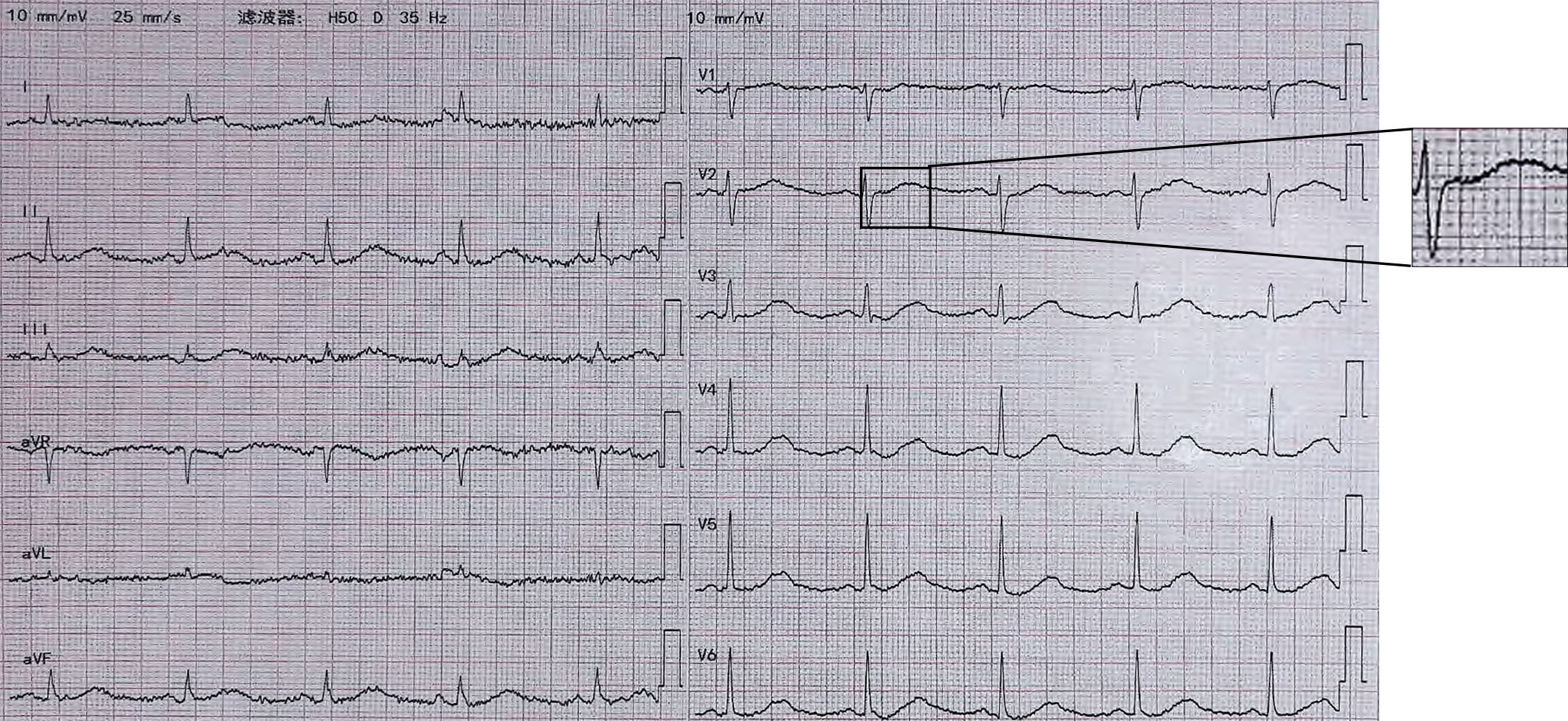

Supplement: Supplementary file 1 [file Datasheet1.zip › 图片/Figure 2.jpg]

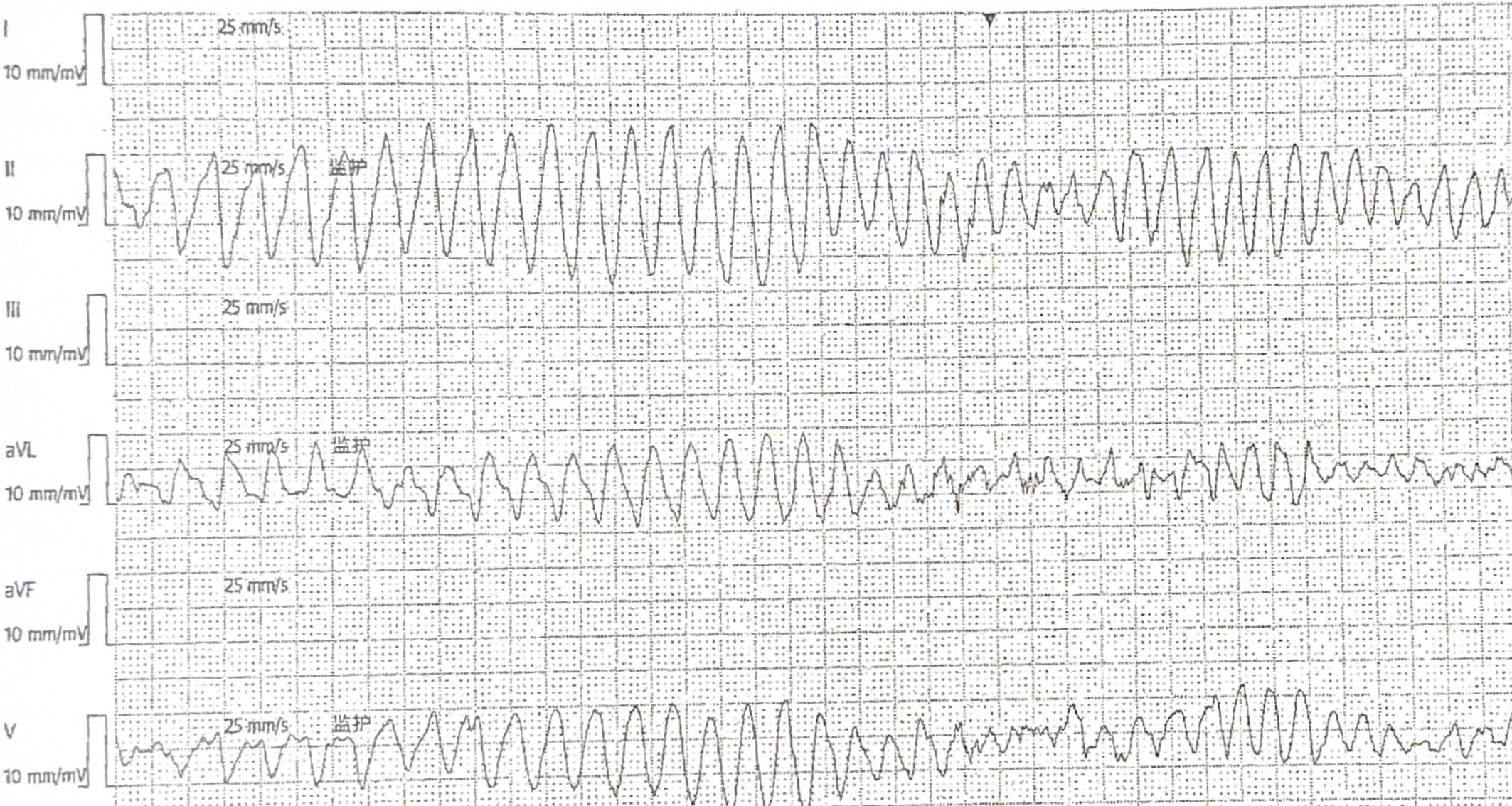

Supplement: Supplementary file 1 [file Datasheet1.zip › 图片/Figure 3.jpg]

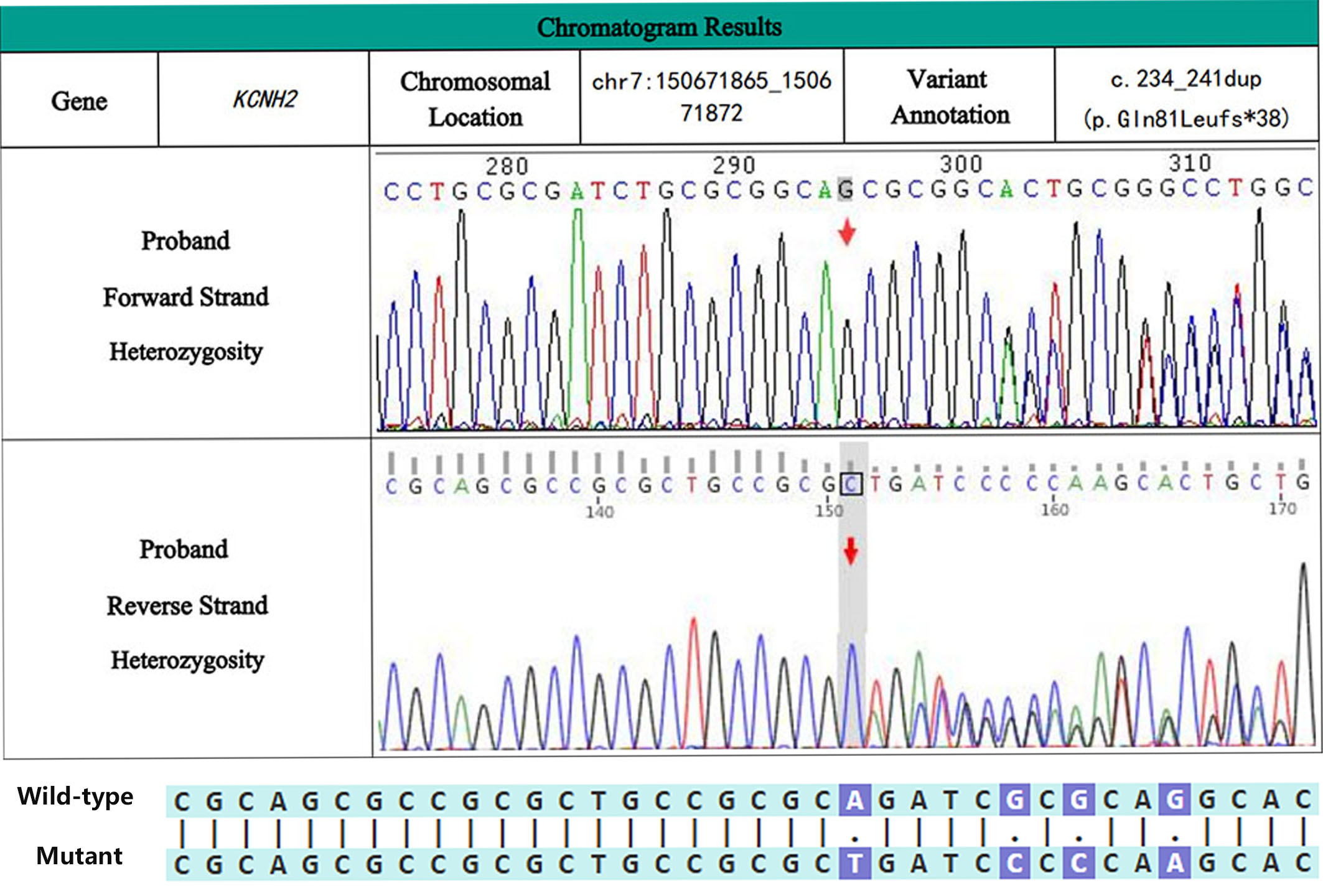

Supplement: Supplementary file 1 [file Datasheet1.zip › 图片/Figure 4.jpg]

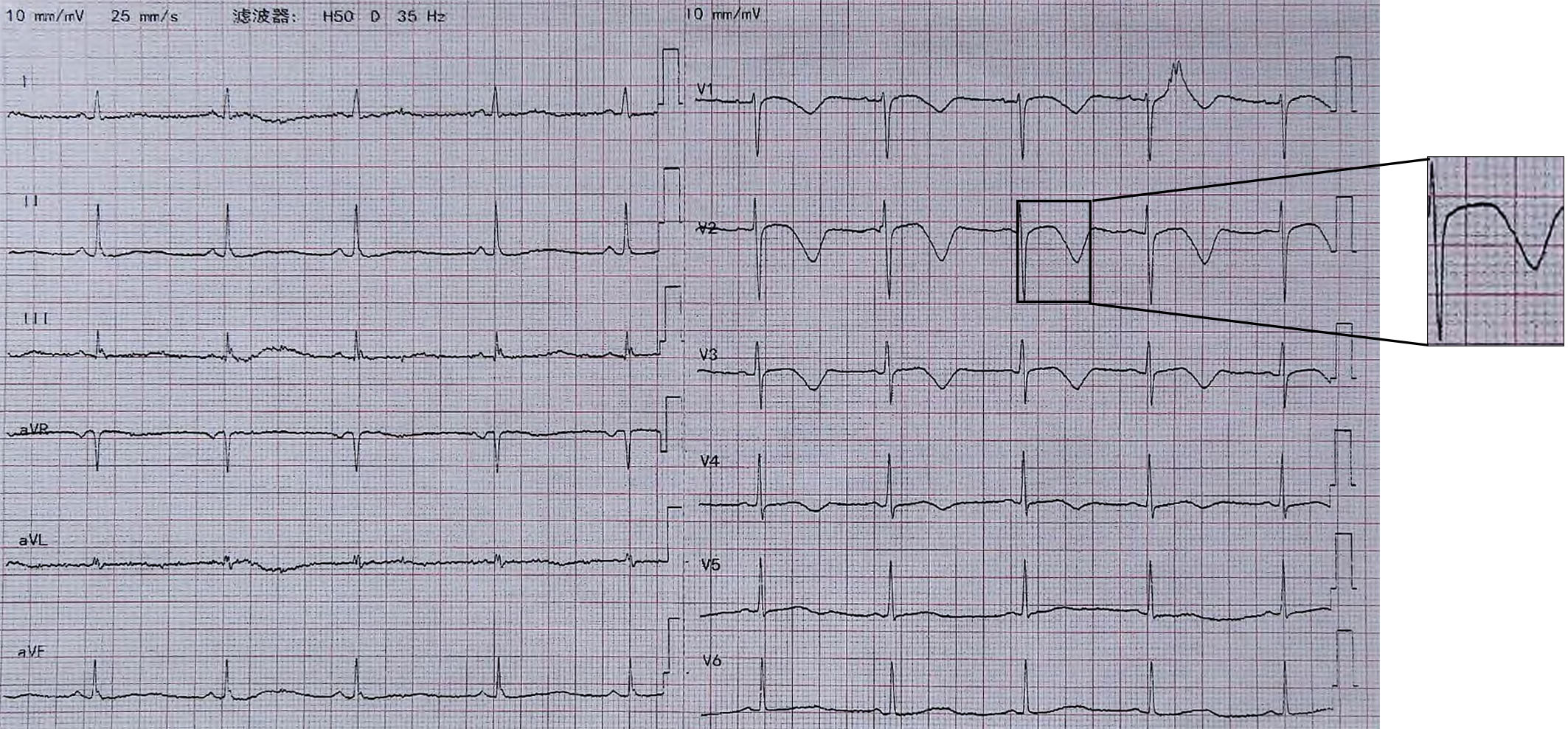

Supplement: Supplementary file 1 [file Datasheet1.zip › 图片/Figure 5.jpg]

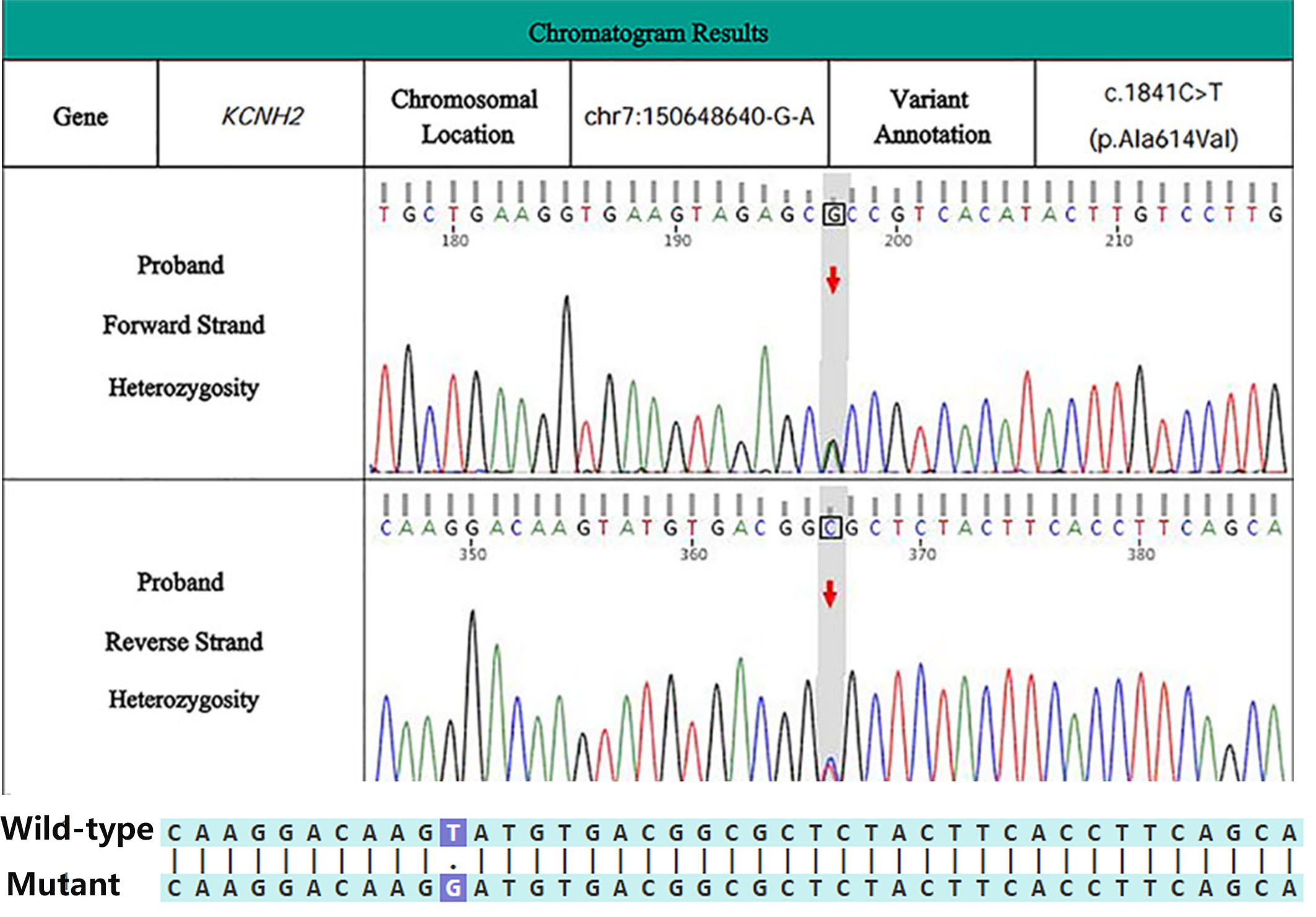

Supplement: Supplementary file 1 [file Datasheet1.zip › 图片/Figure 6.jpg]

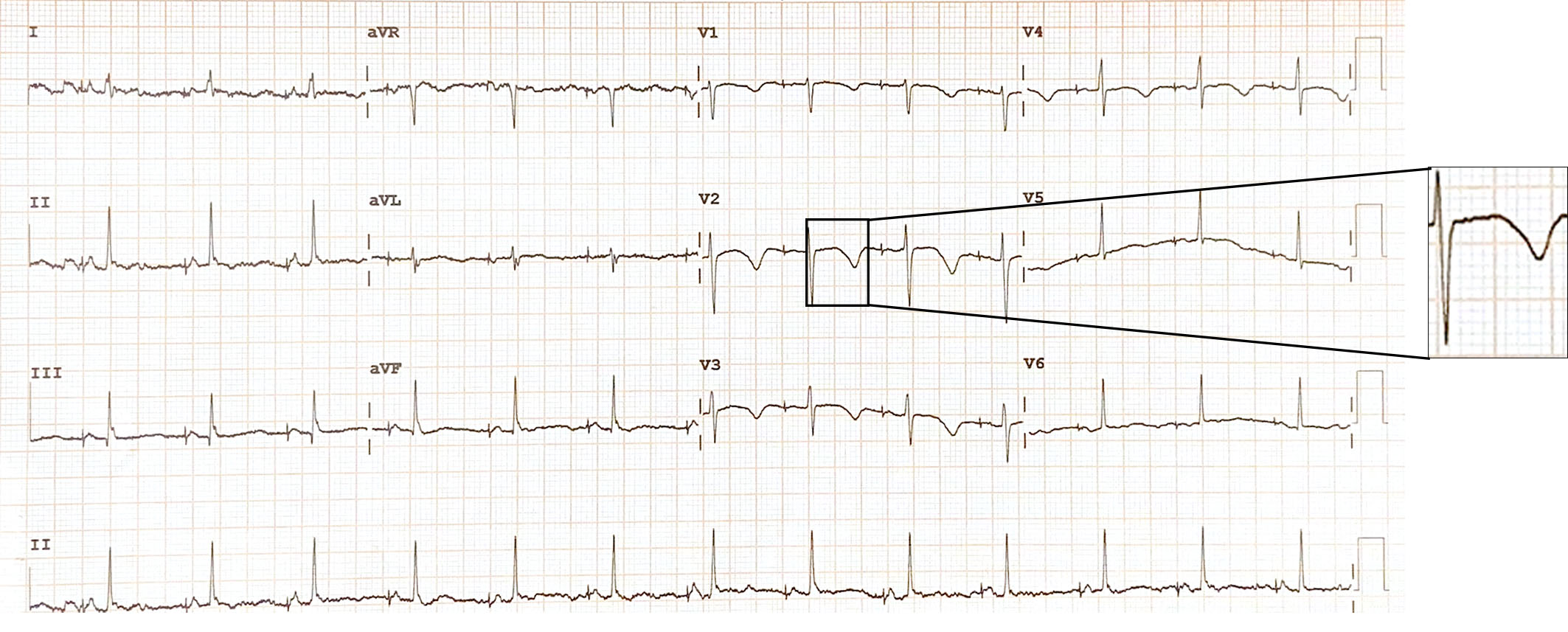

Supplement: Supplementary file 1 [file Datasheet1.zip › 图片/Figure 7.jpg]
